# Supplementary material for: Rosa roxburghii Fruit Extracts Upregulate Telomerase Activity and Ameliorate Cell Replicative Senescence
Source: Foods. 2024 May 27;13(11):1673. doi: 10.3390/foods13111673 (PMC11171777; doi:10.3390/foods13111673)
Supplement: Supplementary file 1 [file foods-13-01673-s001.zip › foods-3021292-supplementary.pptx]

## Slide 1
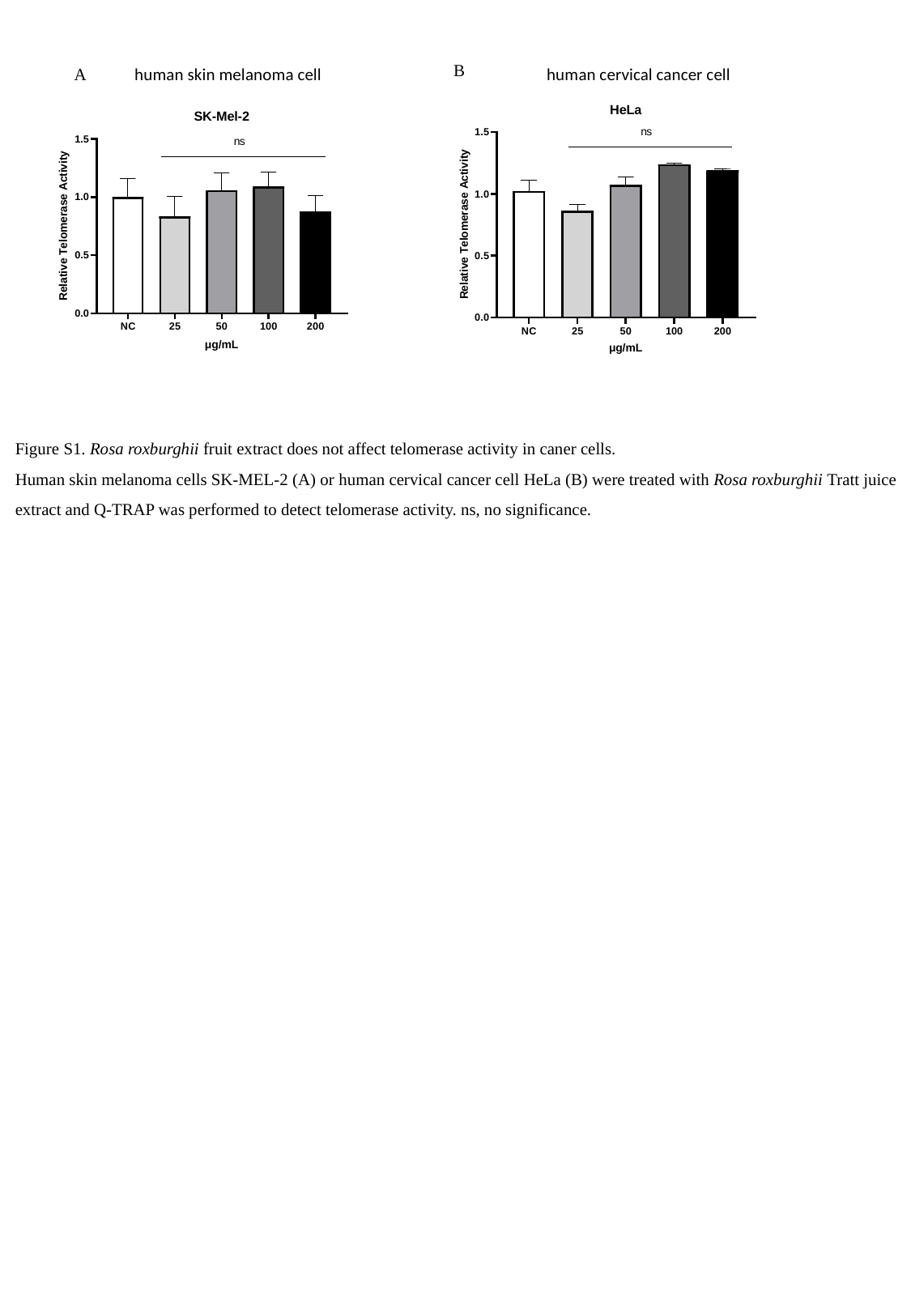

B
human skin melanoma cell
A
human cervical cancer cell
Figure S1. Rosa roxburghii fruit extract does not affect telomerase activity in caner cells.
Human skin melanoma cells SK-MEL-2 (A) or human cervical cancer cell HeLa (B) were treated with Rosa roxburghii Tratt juice extract and Q-TRAP was performed to detect telomerase activity. ns, no significance.

## Slide 2
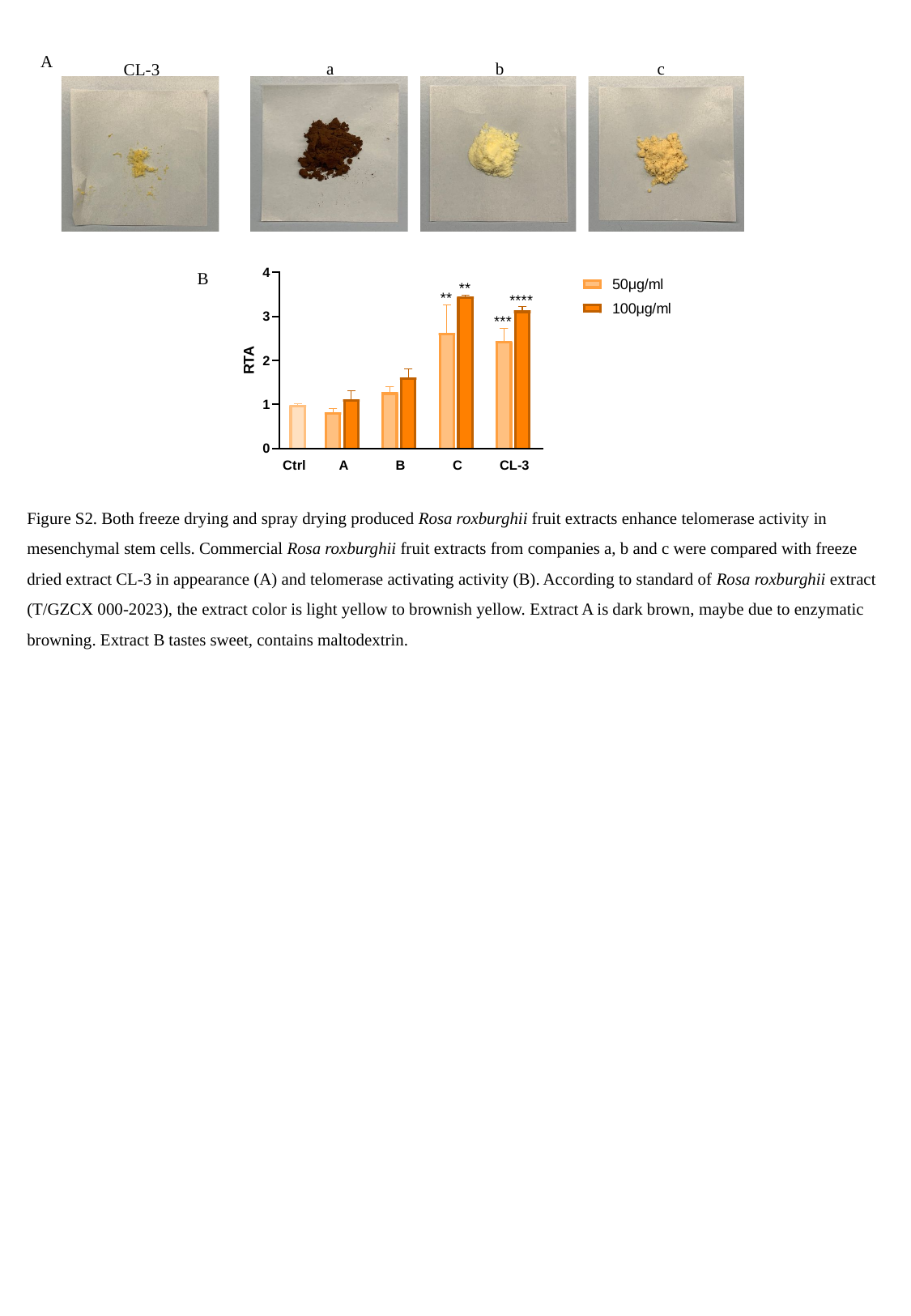

A
a b c
CL-3
B
Figure S2. Both freeze drying and spray drying produced Rosa roxburghii fruit extracts enhance telomerase activity in mesenchymal stem cells. Commercial Rosa roxburghii fruit extracts from companies a, b and c were compared with freeze dried extract CL-3 in appearance (A) and telomerase activating activity (B). According to standard of Rosa roxburghii extract (T/GZCX 000-2023), the extract color is light yellow to brownish yellow. Extract A is dark brown, maybe due to enzymatic browning. Extract B tastes sweet, contains maltodextrin.

## Slide 3
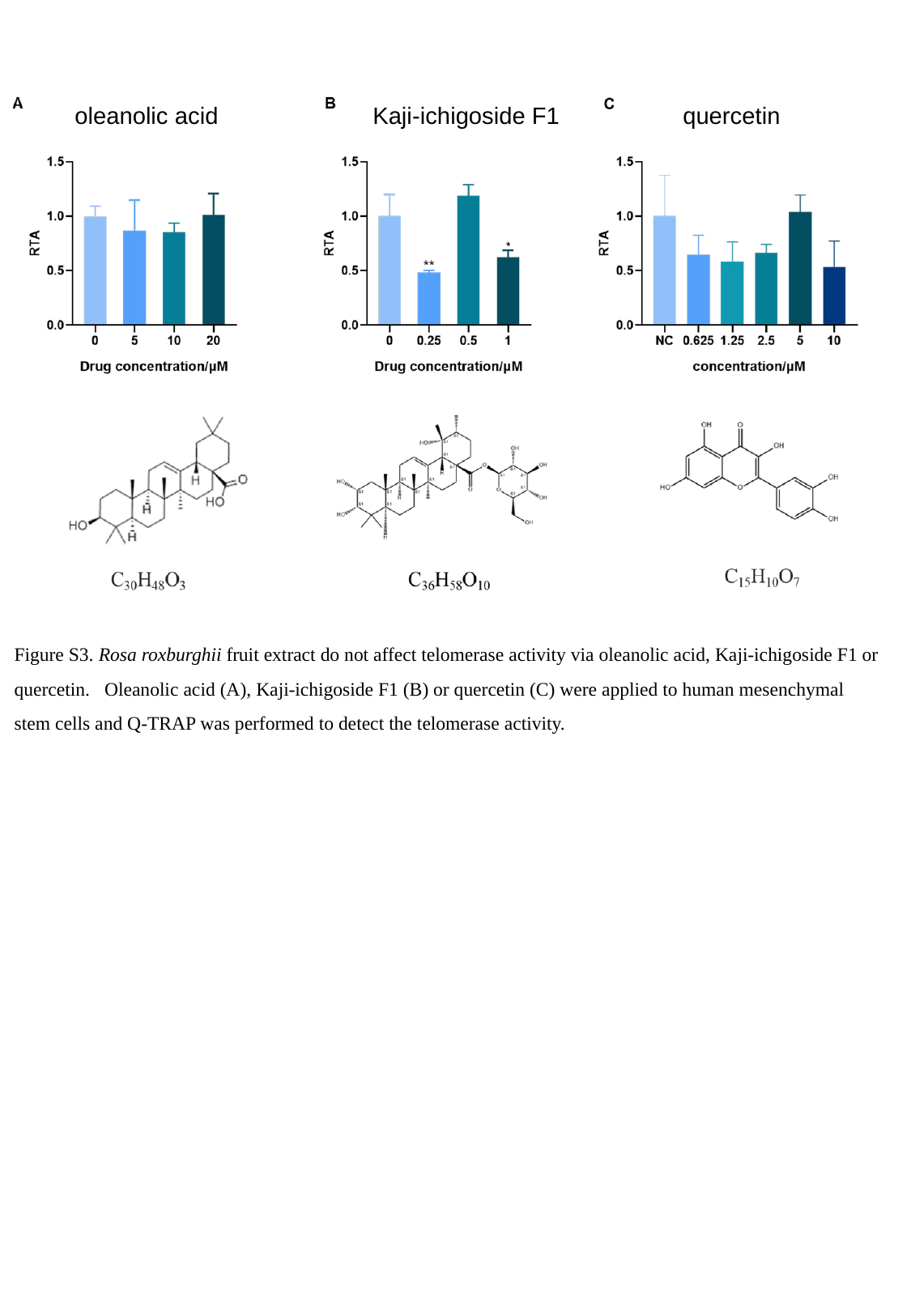

oleanolic acid
Kaji-ichigoside F1
quercetin
Figure S3. Rosa roxburghii fruit extract do not affect telomerase activity via oleanolic acid, Kaji-ichigoside F1 or quercetin. Oleanolic acid (A), Kaji-ichigoside F1 (B) or quercetin (C) were applied to human mesenchymal stem cells and Q-TRAP was performed to detect the telomerase activity.

## Slide 4
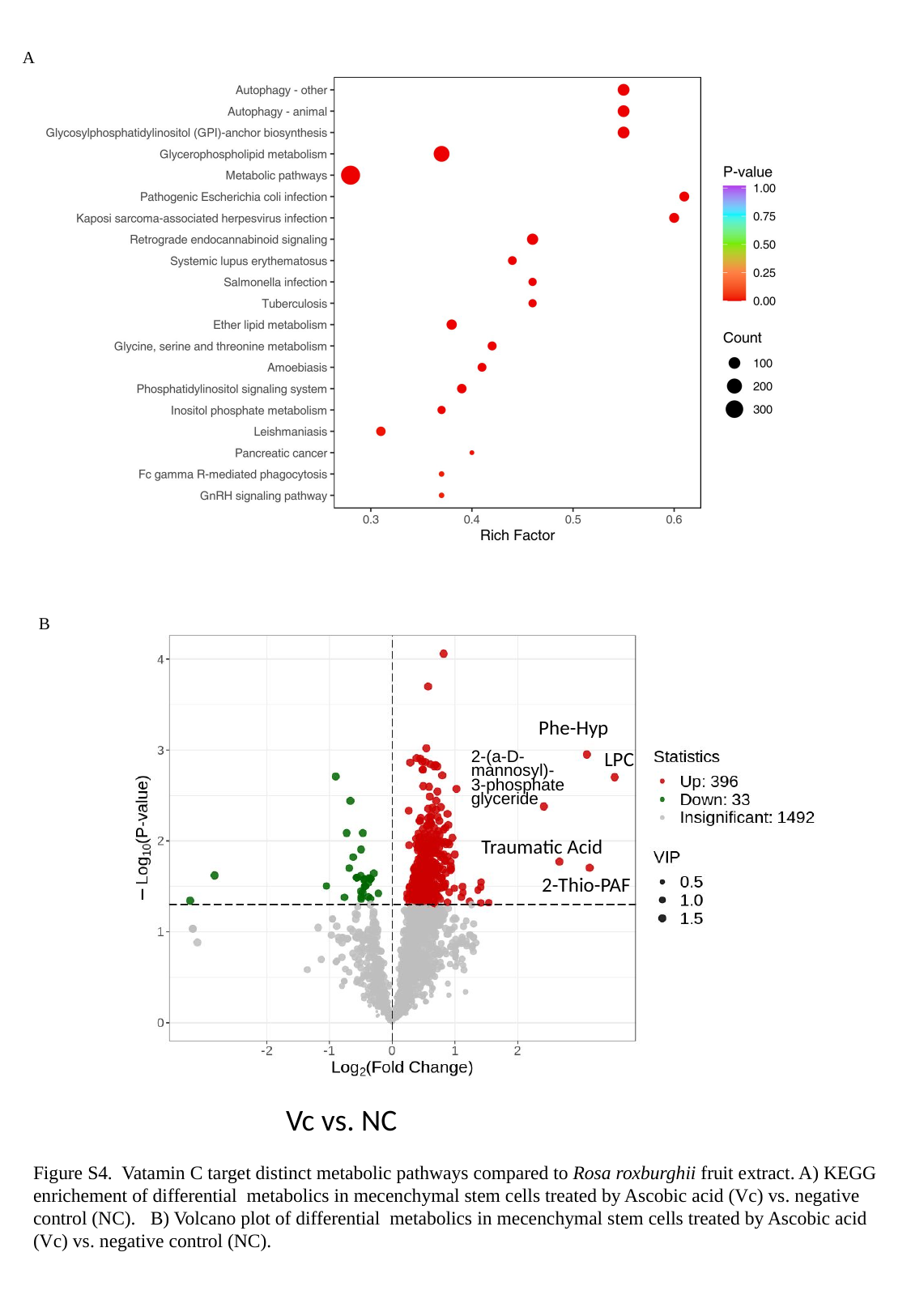

A
B
Phe-Hyp
LPC
2-(a-D-mannosyl)-3-phosphate glyceride
Traumatic Acid
2-Thio-PAF
Vc vs. NC
Figure S4. Vatamin C target distinct metabolic pathways compared to Rosa roxburghii fruit extract. A) KEGG enrichement of differential metabolics in mecenchymal stem cells treated by Ascobic acid (Vc) vs. negative control (NC). B) Volcano plot of differential metabolics in mecenchymal stem cells treated by Ascobic acid (Vc) vs. negative control (NC).
